# Mosquitoes (Diptera: Culicidae) of Poland: An Update of Species Diversity and Current Challenges

**DOI:** 10.3390/insects15050353

**Published:** 2024-05-14

**Authors:** Piotr Jawień, Wolf Peter Pfitzner, Francis Schaffner, Dorota Kiewra

**Affiliations:** 1 Department of Microbial Ecology and Acaroentomology, University of Wroclaw, Przybyszewskiego Str. 63, 51-148 Wrocław, Poland; dorota.kiewra@uwr.edu.pl; 2 KABS e. V., Georg-Peter-Süß Str. 3, 67346 Speyer, Germany; wolf-peter.pfitzner@kabs-gfs.de; 3 Francis Schaffner Consultancy, Lörracherstrasse 50, 4125 Riehen, Switzerland; fschaffner.consult@gmail.com

**Keywords:** mosquito species, Culicidae, Poland, habitat preferences, *Aedes japonicus*, *Anopheles daciae*, *Anopheles hyrcanus*, *Anopheles petragnani*

## Abstract

**Simple Summary:**

This article presents the current state of knowledge of mosquito species occurring in Poland. The current work lists the presence of 51 species of mosquitoes from five genera: *Aedes*, *Anopheles*, *Coquillettidia*, *Culiseta*, and *Culex*, compared to 47 species recorded in works published before 2000. Aspects of the ecology and biology of the Polish mosquito fauna, with particular emphasis on newly recorded species, are discussed.

**Abstract:**

This article presents the current state of knowledge of mosquito species (Diptera: Culicidae) occurring in Poland. In comparison to the most recently published checklists (1999 and 2007), which listed 47 mosquito species, four species (*Aedes japonicus*, *Anopheles daciae*, *Anopheles hyrcanus*, and *Anopheles petragnani*) are added to the Polish fauna. Our new checklist of Polish mosquito fauna includes 51 species of mosquitoes from five genera: *Aedes* (30), *Anopheles* (8), *Coquillettidia* (1), *Culiseta* (7), and *Culex* (5). Aspects of the ecology and biology of the Polish mosquito fauna, with particular emphasis on newly recorded species, are discussed.

## 1. Introduction

Mosquitoes (Diptera: Culicidae) are among the most important vectors of public health importance. Their enormous vector potential for infectious and potentially lethal pathogens and parasites makes them the most important blood-feeding arthropods in the world. Climate and land-use changes, globalization, and intensive transportation of humans and goods influence the spread of mosquitoes and pathogens, which results in expanding emerging or re-emerging diseases around the world [1,2,3]. Knowledge of the presence and current range of mosquito species occurrence in a given area is therefore crucial to assessing the potential risk to human and animal health. This paper aims to present an up-to-date state of knowledge of the mosquito fauna in Poland. The previous checklist of mosquito species occurring in Poland was published at the end of the 20th century by Kubica-Biernat [4], who listed in her publication 47 mosquito species reported from 223 identifiable sites in Poland. The same list of 47 species was also given by Wegner in 2007 [5].

## 2. Current Knowledge

The knowledge of the distribution of Culicidae in Poland still remains fragmentary because regular monitoring of the presence of mosquito species is limited to only a few areas. Additionally, some data on mosquito species occurrence are included in papers concerning biocontrol [6,7,8], evening activity of synanthropic species [9], factors influencing the distribution of floodwater mosquito eggs [8], and the detection of pathogens [10,11,12,13]. 

**Figure 1:**
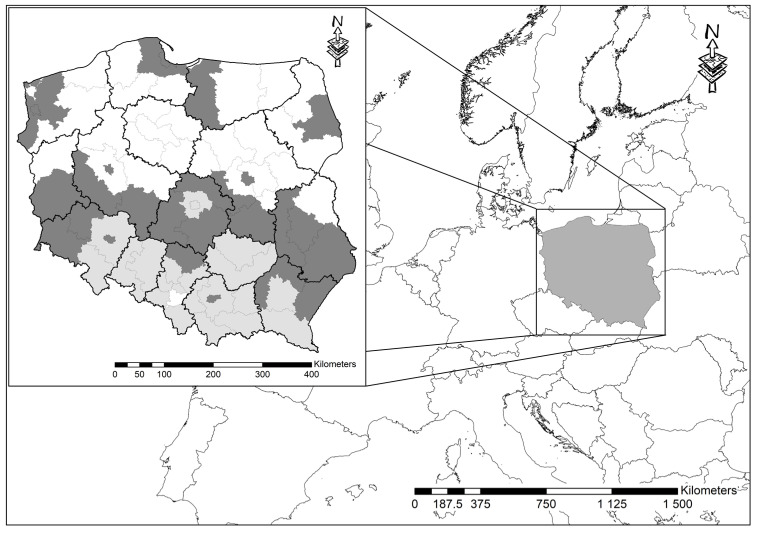
NUTS 3 territorial units in Poland, where work on the species composition of mosquitoes was carried out between the years 2000–2023 (dark grey), light grey areas—units where *Ae. japonicus* was found in 2023 [29,30].

Original articles on the occurrence of mosquitoes in Poland published after 2000 come from 15 out of 16 voivodeships (Figure 1, Table 1, Table 2 and Table 3) and concern mostly data from urban areas, including Wrocław [5,10,14,15,16,17,18,19,20], Gdańsk, Gdynia, Sopot [21], Krynica Morska [22], Warsaw [23], Poznań [24], and Świnoujście [25]. Additionally, based on literature data, a review of species occurring in five Polish towns (Szczecin, Świnoujście, Gdańsk, Warszawa, Wrocław) was published [26]. Scarce data concern protected areas such as the Narew National Park [27] and the Iława Lakeland Landscape Park [28]. Finally, data on mosquito distribution come from monitoring data from south regions of Poland published by the ECDC [29] and collected during a VectorNet capacity-building program [30].

In recent years, records of mosquito species newly found in Poland have been published. Species not mentioned in the review by Kubica-Biernat [4] and described in original papers published after 2000 include three species of *Anopheles*: *An. hyrcanus* [20], *An. daciae* [19], and *An. petragnani* [30]. Additionally, in 2023, *Ae. japonicus* was found in southern Poland [29,30].

The unequivocal evidence of *An. daciae*’*s* presence in Poland was shown for the first time by Rydzanicz et al. [19]. They collected adult mosquitoes between July and September 2015 in the Wrocław area and applied a rapid PCR-RFLP assay on specimens of the Maculipennis Complex, resulting in the unambiguous identification of *An. daciae*, *An. messeae*, and *An. maculipennis sensu stricto* (*s.s.*). The potential occurrence of *An. daciae* was also suggested earlier by Kubica-Biernat and Kowalska-Ulczyńska [31] in north and north-eastern Poland (the voivodeships of Pomorskie, Warmińsko-Mazurskie, and Podlaskie). Here, *An. maculipennis s.s*. and the taxon *An. messeae/daciae* were identified among over a thousand mosquito specimens of the Maculipennis Complex. *Anopheles messeae* and *An. daciae* are morphologically and genetically almost similar, which challenges species identification. Both species prefer stagnant water bodies with unpolluted water on the banks of rivers and lakes with abundant submerged vegetation, ponds, and ditches, as well as larger water bodies in floodplains (Table 4)*. Anopheles daciae* was originally distinguished from *An. messeae* based on five nucleotide substitutions in its rDNA internal transcribed spacer 2 [32]. *Anopheles daciae* occurs in Eurasia, including countries neighboring Poland. It was found in Germany [33,34,35,36], Czechia, and Slovakia [37,38], as well as in Finland [39] and Russia [40,41].

The first report on the appearance of *An. hyrcanus* in Poland comes from Wrocław (southwest Poland), where this species was identified among mosquitoes collected in the years 2019–2020 [20]. The identification of a well-established population of *An. hyrcanus* in Wrocław [20] suggests an increase in the range of this species because the described place of its occurrence is the most northern localization in Europe. *Anopheles hyrcanus sensu lato* is considered to be a species complex that includes about 30 species in the Palearctic and Oriental regions [42,43]. In the Wrocław area, *An. hyrcanus s.s.* was discovered in wetlands containing numerous canals and permanent reservoirs situated in the valleys of the Odra and Oława rivers. This region is a natural area covered with riparian forests where white willow (*Salix alba*) and white poplar (*Populus alba*) are the dominant species. It is also recognized as a protected region under the European network of Natura 2000 areas and is covered by a sanitary protection zone [14]. The banks of canals, lakes, and wet areas are covered with marsh vegetation—reeds and sedge communities with common sedge (*Carex fusca*), sharp sedge (*Carex gracilis*), common calamus (*Acorus calamus*), and common reed (*Phragmites australis*). The preference of habitat for *An. hyrcanus* in Wrocław is in line with data presented by Becker et al. [3], who described that the larvae of *An. hyrcanus* prefer to develop in more or less clear, sun-exposed water bodies with rich vegetation. The larvae of *An. hyrcanus* mosquitoes can be found in permanent reservoirs and slowly flowing channels that have rich riparian plants (Table 4). The presence of *An. hyrcanus* was documented in many European countries, including France [44], Croatia, Hungary, Austria [45], Czechia, Slovakia, Russia [43,46,47], Romania, Serbia [48], Italy [49], and Spain [44].

*Anopheles petragnani* was very recently found for the first time in Poland in the Kielce Upland [30]. Larvae were collected in artificial water reservoirs (metal wadies) in allotment gardens in Kielce. The species is a sibling species of *Anopheles claviger s.s.*, whose morphological differentiation is only possible on the basis of subtle differences in the structure of eggs and larvae [50,51]. PCR methods are used to unambiguously distinguish these species [52]. *Anopheles petragnani* is distributed in the western Mediterranean [3], whose larvae tolerate higher water temperatures than *An. claviger s.s*. [50]. It is a zoophilic species capable of autogenous reproduction. In the past, it was sporadically recorded in southern Europe (Portugal, Spain, southern France, Italy, and North Africa) and more recently in Germany [51] and Luxembourg [53]. It does not play any role in pathogen transmission [3].

*Aedes japonicus* was found in the southern part of Poland during a capacity building program organized by ECDC [29,30]. All positive records are from southern regions close to the border of Czechia and Slovakia. In Czechia *Ae. japonicus* was found in 2021, close to the Czech–German and Czech–Austrian border [54] and recently in the north of the country [30]. In Slovakia, the first records of *Ae. japonicus* were found in 2020 in a few places localized at border crossings with Austria and Hungary and in the urban and rural zones of five major cities [55]. In Germany, *Ae. japonicus* was found for the first time in 2008 in the south German federal state of Baden-Wuerttemberg [56,57,58] and is now widespread over Germany [59]. *Aedes japonicus* is a species native to Asia (Korea, Japan, Taiwan, southern China, and south-eastern Siberia), and it is recognized as the third invasive mosquito species reported in Europe, after *Ae. albopictus* and *Ae. aegypti* [2]. 

The mosquito species *Ae. rossicus* appears here for the first time at the species rank in a Polish checklist, while it was listed as a subspecies of *Ae. esoensis* in the review of Kubica-Biernat [4]. Indeed, this taxon is nowadays recognized as a valid species [60]. In recent two decades, the presence of *Ae. rossicus* was noted in Warsaw (Wilanów) in a study that listed 33 mosquito species (26 species recorded as larvae and 30 as adults) recorded in the years 2001–2009 in material that comprised more than 11,000 individuals, including larvae and adults of *Ae. rossicus* [23]. Furthermore, *Ae. rossicus* was recorded in Warsaw for the first time. *Aedes rossicus* is a floodwater mosquito that lays eggs on the ground that is frequently flooded; it prefers small temporary water bodies, inundation areas of river valleys, and swampy woodlands with acid waters (Table 4). The presence of *Ae. rossicus* was also confirmed in other European countries, including Sweden [61], France [62], Germany [63], Czechia [64], and Russia [60,65].

Among the 47 mosquito species listed by Kubica-Biernat [4], during the last two decades, the presence of 8 species was not confirmed: *Ae. detritus*, *Ae. hexodontus*, *Ae. nigripes*, *Ae. pionips*, *Ae. pullatus*, *An. atroparvus*, *Cs. fumipennis*, and *Cs. subochrea* (Table 1, Table 2 and Table 3). The occurrence of *Ae. geminus* was recently confirmed [20] in Lower Silesia [30]. The smaller number of mosquito species identified in recent years is most likely the result of the lack of monitoring in most areas of Poland and might not be related to the disappearance of species. It is worth emphasizing that the mosquito fauna in Poland includes species with different habitat preferences (Table 4). For development, they prefer different types of water bodies (e.g., clear or highly polluted); some species use inland brackish marshes and other snow-melt ponds [3,8,66,67]. They can hatch in wild and synanthropic areas, in permanent and temporary water bodies, and in different types of water bodies along rivers, including flooded areas in river valleys. Species also differ in their development time (spring—most of them, or summer), number of generations (mono- or polycyclic), as well as host feeding preferences (anthropo- or zoophilic), and vector potential. Thus, mosquito fauna studies should cover diverse environments and different geographical areas to be comprehensive.

## 3. Medical Importance

Among the mosquito species found in Poland, 40% are potential or documented vectors of pathogens (Table 4) [2,20,26,68]. These species can transmit malaria plasmodia (currently in Poland, only imported malaria cases are reported), arboviruses, or zoonotic parasites. Among them, mosquito species recorded in Poland, *Ae. vexans* and *Cx. pipiens s.l*. are of particular importance because they are widespread throughout the country and have documented vector roles. They are multi-voltine species that can thrive in large numbers under favorable conditions.

*Aedes vexans* is a floodwater species common in the Holarctic region, showing high abundance in Eastern Europe [3]. It thrives in a variety of habitats, particularly in rural areas, and is commonly found in floodplains of river valleys. Like many floodplain mosquitoes, *Ae. vexans* prefer to lay eggs on the ground of temporary or semi-permanent pools that are prone to seasonal flooding. Their eggs enter a state of diapause, which allows them to survive longer periods of drought before hatching in large numbers after floods. *Aedes vexans* has an extremely short aquatic phase in summer (6–7 days), which leads to a rapid increase in population density. Adult females are known for aggressive biting behavior, showing little host specificity among mammals and humans. This feature is important for the potential transmission of pathogens. In regions such as North America and Europe, *Ae. vexans* transmits a variety of arboviruses, including West Nile virus (WNV), snow hare virus (SSHV), Jamestown Canyon virus (JCV), Tahyna virus (TAHV), and Batai virus (BATV). In Africa, *Ae. vexans* is considered one of the main vectors of the Rift Valley fever virus (RVFV). Experimental studies have confirmed its ability to transmit RVFV in both African and American populations [68,69].

The widespread distribution and the preference for urban environments, close to people make *Cx. pipiens* a significant concern for public health worldwide [68,70,71,72,73]. This species complex is known for transmitting pathogens such as WNV, Eastern equine encephalitis virus (EEEV), St. Louis encephalitis virus (SLEV), Sindbis virus (SINV), Usutu virus (USUV), BATV, and filarial worms such as *Dirofilaria repens* or *Dirofilaria immitis* [10].

From a medical point of view, the emergence of invasive mosquito species may be of particular importance because some of them play a key role in the spread of many mosquito-borne diseases worldwide [2]. In Europe, the most important invasive mosquito species include: *Ae. albopictus*, *Ae. aegypti*, *Ae. japonicus*, and *Ae. koreicus.* In Poland, so far only the presence of *Ae. japonicus* has been confirmed; however, the presence of other invasive species (*Ae. albopictus*, *Ae. koreicus*) is increasingly noted in countries bordering Poland [74,75,76,77]. *Aedes japonicus*, native to Japan, Korea, Taiwan, southern China, and parts of Russia, did spread to North America and Central Europe. *Aedes japonicus* shows early emergence and a longer activity season compared to other invasive species, as well as the ability to disperse over long distances and to withstand lower temperatures. However, it cannot tolerate aquatic habitats exceeding 30 °C, limiting its spread in Southern Europe. Similarly to *Ae. albopictus*, it produces cold-resistant eggs and feeds on mammals primarily. It adapts well to various environments, including urban areas with artificial containers, but prefers vegetation-rich suburban or rural settings and agricultural environments. Its minimal habitat requirements and tolerance to organic matter facilitate its spread even in eutrophic and polluted water basins. *Aedes japonicus* has been linked to outbreaks of the Japanese encephalitis virus (JEV) in Asia and to La Crosse virus and WNV cycles in North America [59]. Its presence in new areas has led to an increase in its own abundance and the displacement or reduction in native mosquitoes, which may affect mosquito diversity as well as the epidemiology of associated arboviruses. Although not very widespread yet, there is concern that the range and abundance of this species will expand in Poland [78]. 

The southern part of Poland appears to be suitable for the emergence of further invasive mosquito species due to higher average temperatures than in other areas of the country and the proximity of invasive mosquito populations in neighboring countries. Therefore, it is necessary to continuously monitor and survey mosquito species composition along known transmission routes—motorways, airports, etc. Understanding the biology and ecology of native and invasive mosquito species is essential for developing strategies to prevent and control the spread of mosquito-borne diseases. Furthermore, the rapid process of globalization, coupled with climate change, which affects mosquito populations, highlights the need for capacities for applying effective vector control measures to mitigate pathogen transmission.

## 4. Conclusions

The current checklist of mosquito species in Poland includes 51 species of five genera: *Aedes*, *Anopheles*, *Coquillettidia*, *Culiseta*, and *Culex.* It is worth emphasizing that two first records (*An. daciae* and *An. hyrcanus*) were located in Wrocław—an area under regular mosquito monitoring [19,20]. In addition, *Ae. japonicus* was found to be well established in southern Poland in seven voivodships, in 2023, after remaining undetected for several years, probably due to the lack of earlier monitoring in these areas [30]. Surveys conducted in 2023 also resulted in the identification of *An. petragnani* in central Poland, in the Kielce Upland [30]. Both species were found in the country for the first time ever. Considering the ecological diversity of mosquito species occurring in Poland, and the limited area of mosquito fauna monitoring conducted in recent years, the accurate knowledge of the distribution of individual mosquito species in Poland requires further, comprehensive research to be conducted throughout the country. Research conducted during the last few decades focused mainly on urban areas. It would be worthwhile in the future to extend monitoring and research of the mosquito fauna to rural and natural areas. Moreover, due to the emergence of invasive species such as *Ae. japonicus*, *Ae. albopictus*, and *Ae. koreicus* in countries bordering Poland [74,75,76,77], it seems necessary to continue to track changes at the border of their range of occurrence and further investigate the possible occurrence of invasive mosquito species in Poland. 

Globalization and climate change around the world and in Europe will favor the further spread of mosquitoes to new areas. Invasive species such as *Ae. albopictus* are a good example. The emergence of additional mosquito species raises challenges in terms of eliminating introduced specimens or suppressing established populations over larger areas. Additionally, invasive species are often competent vectors of disease pathogens that can emerge subsequently. Preventing or controlling mosquito-borne diseases will be a major challenge for the health services of many countries, not least for Poland, which has little mosquito control experience and capacity and is unprepared for facing mosquito-borne diseases.

**Table 1:** The occurrence of *Aedes* mosquitoes in Poland.

Country/ Voivodeship	Authors	*Ae. annulipes*	*Ae. behningi*	*Ae. cantans*	*Ae. caspius*	*Ae. cataphylla*	*Ae. cinereus*	*Ae. communis*	*Ae. cyprius*	*Ae. detritus*	*Ae. diantaeus*	*Ae. dorsalis*	*Ae euedes*	*Ae. excrucians*	*Ae. flavescens*	*Ae. geminus*	*Ae. geniculatus*	*Ae. hexodontus*	*Ae. intrudens*	*Ae. japonicus*	*Ae. leucomelas*	*Ae. nigrinus*	*Ae. nigripes*	*Ae. pionips*	*Ae. pullatus*	*Ae. punctor*	*Ae. riparius*	*Ae. rossicus*	*Ae. rusticus*	*Ae. sticticus*	*Ae. vexans*
**Published until 1999**																															
Poland	[4]	+	+	+	+	+	+	+	+	+	+	+	+	+	+	+	+	+	+		+	+	+	+	+	+	+	+	+	+	+
**Published after 2000**																															
Wielkopolskie	[11]	+		+			+																			+				+	+
	[24]	+		+																										+	+
	[13]	+		+				+																		+				+	+
Dolnośląskie	[14]			+				+						+																+	+
	[15]			+				+						+																+	+
	[79]	+		+	+	+	+	+				+			+		+				+									+	+
	[19]	+		+	+		+																							+	+
	[8]				+																										+
	[7]				+																										+
	[17]				+																										+
	[18]			+	+																+									+	+
	[10]			+	+		+																							+	+
	[20]	+					+ *									+ *	+											+		+	+
	[30]																+			+											+
Mazowieckie	[23]	+		+	+	+	+	+	+		+	+	+	+	+		+		+		+	+				+	+	+	+	+	+
	[30]																+														
Podlaskie	[27]	+		+		+	+	+					+	+	+				+		+					+	+			+	+
Pomorskie	[80]	+		+	+	+	+	+						+			+		+		+	+				+				+	+
	[21]	+		+	+	+	+	+							+		+				+					+				+	+
	[22]	+			+	+		+						+			+		+		+									+	
Warmińsko-Mazurskie	[81]	+		+		+	+	+				+		+	+				+		+					+				+	+
	[28]	+	+	+	+ **	+	+	+				+ **	+	+	+		+		+		+	+				+	+			+	+
Zachodniopomorskie	[25]	+		+	+		+	+																		+				+	+
Łódzkie	[30]	+ ***		+ ***			+ *									+ *				+											+
Lubelskie	[30]																													+	+
Małopolskie	[30]						+ *									+	+			+											+
Opolskie	[30]																+			+										+	
Podkarpackie	[30]																			+											
Śląskie	[30]	+ ***		+ ***			+ *										+			+										+	+
Świętokrzyskie	[30]	+ ***		+ ***																+											

* Species identified as *Aedes cinereus/geminus*; ** species identified as *Aedes caspius/dorsalis*; *** species identified as *Aedes annulipes/cantans*.

**Table 2:** The occurrence of *Anopheles* mosquitoes in Poland.

Country/Voivodeship	Authors	*An. atroparvus*	*An. claviger* s.s.	*An. daciae*	*An. hyrcanus*	*An. maculipennis* s.l. ^#^	*An. maculipennis* s.s.	*An. messeae*	*An. plumbeus*	*An. petragnani*
**Published until 1999**										
Poland	[4]	+	+			+	+	+	+	
**Published after 2000**										
Wielkopolskie	[13]							+		
Dolnośląskie	[14]					+				
	[15]					+				
	[79]					+				
	[18]					+				
	[19]		+	+		+	+	+	+	
	[10]		+			+				
	[82]					+				
	[20]		+ **		+	+			+	
	[30]		+ **				+		+	
Mazowieckie	[23]		+			+			+	
	[30]								+	
Podlaskie	[31]			+ *			+	+ *		
	[27]					+				
Pomorskie	[80]		+			+				
	[31]			+ *			+	+ *		
	[21]		+			+				
	[50]		+							
Warmińsko-Mazurskie	[81]		+			+		+		
	[28]		+			+		+	+	
	[31]			+ *			+	+ *		
Zachodniopomorskie	[25]					+				
Łódzkie	[30]		+				+		+	
Lubelskie	[30]		+			+			+	
Małopolskie	[30]		+	+			+		+	
Opolskie	[30]								+	
Podkarpackie	[30]						+		+	
Śląskie	[30]		+					+	+	
Świętokrzyskie	[30]									+

* Species identified as *An. messeae/daciae*; ** species identified as *Anopheles claviger/Anopheles petragnani*; *^#^* the complex of several species not distinguished in many works.

**Table 3:** The occurrence of *Coquillettidia*, *Culiseta*, and *Culex* mosquitoes in Poland.

Country/Voivodeship	Authors	*Cq. richiardii*	*Cs. alaskaensis*	*Cs. annulata*	*Cs. fumipennis*	*Cs. glaphyroptera*	*Cs. morsitans*	*Cs. ochroptera*	*Cs. subochrea*	*Cx. hortensis*	*Cx. modestus*	*Cx. pipiens* s.l.	*Cx. territans*	*Cx. torrentium*
**Published until 1999**														
Poland	[4]	+	+	+	+	+	+	+	+	+	+	+ *	+	+
**Published after 2000**														
Wielkopolskie	[11]	+												
	[24]	+												
	[12]	+										+ *		+
	[13]	+										+ *	+	
	[30]			+								+ **		
Dolnośląskie	[14]			+								+ *		
	[15]			+								+ *		
	[9]			+								+ *		
	[79]	+	+	+								+		
	[66]											+ *		+
	[19]	+		+								+ *		+
	[6]											+		
	[16]											+ **		+ **
	[17]											+		
	[18]			+								+ *		+
	[83]											+ *		+
	[10]	+		+								+ **		+ **
	[82]											+ *		+
	[20]	+		+								+ **		+ **
	[30]					+						+, + **	+	+ **
Mazowieckie	[23]	+	+	+				+			+	+	+	+
	[30]			+						+		+, + **		+ **
Podlaskie	[27]	+	+	+								+ *	+	+
Pomorskie	[80]	+	+	+			+					+ *	+	
	[21]	+		+			+					+ *	+	+
	[22]			+			+						+	
Warmińsko-Mazurskie	[81]	+	+	+			+					+ *	+	+
	[28]	+	+	+			+	+				+ *	+	+
Zachodniopomorskie	[25]	+		+							+	+ **		+ **
Łódzkie	[30]			+								+, + **	+	+ **
Lubelskie	[30]			+								+, + **	+	+ **
Małopolskie	[30]			+								+, + **		+, + **
Opolskie	[30]			+								+		+
Podkarpackie	[30]			+								+, + **		+ **
Śląskie	[30]	+										+ **	+	+ **
Świętokrzyskie	[30]											+, + **		+ **

* *Cx. pipiens* without recognition of biotype; ** *Culex pipiens/torrentium*.

**Table 4:** Mosquito species found in Polish fauna—their characteristics and habitat preferences [3,67].

No.	Species	Number of Generations	Emerging Period	Host Preference	Vector Potential	Habitat Preferences [3,67]
1.	*Aedes* (*Ochlerotatus*) *annulipes* (Meigen, 1830)	M	Sp	A	+	open meadow pools, forest edges, and deciduous forests, semipermanent reservoirs with leaf detritus
2.	*Aedes* (*Ochlerotatus*) *behningi* (Martini, 1926)	M	Sm	A		flooded areas on river valleys
3.	*Aedes* (*Ochlerotatus*) *cantans* (Meigen, 1818)	M	Sp	A	+	open permanent or semipermanent meadow pools, deciduous or mixed forest reservoirs with scarce aquatic vegetation and layer of leaves on the bottom
4.	*Aedes* (*Ochlerotatus*) *caspius* (Pallas, 1771)	P	Sm	A	+	inland salt marshes, fresh waters, coastal marshes and rock holes, open or shaded waters, permanent or temporary snow-melt water bodies, and after floods, river floodplains
5.	*Aedes* (*Ochlerotatus*) *cataphylla* Dyar, 1916	M	Sp	A, Z		forest pools in swampy woodlands, reservoirs with dead leaves on the bottom, inundated meadows, neutral to alkaline water
6.	*Aedes* (*Aedes*) *cinereus* Meigen, 1818	P	Sp	A	+	semipermanent water bodies, flood plains, sedge marshes with *Sphagnum* sp., bogs, lakes covered by emerged vegetation, woodland pools
7.	*Aedes* (*Ochlerotatus*) *communis* (De Geer, 1776)	M	Sp	Z		snow-melt forest reservoirs, acidic waters, ditches, and reservoirs with dead leaves on the bottom
8.	*Aedes* (*Ochlerotatus*) *cyprius* Ludlow, 1920	M	Sp	Z	+	semipermanent reservoirs along inundated river shores, snow-melt pools
9.	*Aedes* (*Ochlerotatus*) *detritus* (Haliday, 1833)	M	Sp	A		meadows, reservoirs with an exceptionally high content of salinity, brackish waters, and coastal marshes, semipermanent ponds with *Salicornia* sp. and *Tamarix* sp. marshes, stagnant drainage channels with saline water
10.	*Aedes* (*Ochlerotatus*) *diantaeus* (Howard, Dyar and Knab, 1913)	M	Sp	Z		temporary open water bodies formed after snow-melt with dead leaves at the bottom, shaded ditches, and pools in mixed forests
11.	*Aedes* (*Ochlerotatus*) *dorsalis* (Meigen, 1830)	P	Sp	A	+	small open water bodies, swamps, permanent and temporary water reservoirs on inland pastures formed by melted snow, floods, rainfall or groundwater, roadside water bodies, and drainage ditches, also saline waters
12.	*Aedes* (*Ochlerotatus*) *euedes* Howard, Dyar and Knab, 1913	M	Sp	A		open permanent or semipermanent meadow pools, deciduous or mixed forest reservoirs with scarce aquatic vegetation and layer of leaves on the bottom
13.	*Aedes* (*Ochlerotatus*) *excrucians* (Walker, 1856)	M	Sp	A		shaded permanent water bodies, open semi-permanent or permanent reservoirs with vegetation like *Typha* sp. or *Carex* sp., mixed forests
14.	*Aedes* (*Ochlerotatus*) *flavescens* (Müller, 1764)	M	Sp	A		reeded areas of water bodies, brackish marshes along coasts, shaded temporary reservoirs in floodplains, neutral to slightly alkaline water, and a wide range of salinity
15.	*Aedes* (*Aedes*) *geminus* Peus, 1970	P	Sp	A		semipermanent water bodies, flood plains, sedge marshes with *Sphagnum* sp., bogs, lakes covered by emerged vegetation, woodland pools
16.	*Aedes* (*Dahliana*) *geniculatus* (Olivier, 1791)	P	Sp	A	+	deciduous forests, parks, open tree stumps, tree holes of deciduous trees
17.	*Aedes* (*Ochlerotatus*) *hexodontus* Dyar, 1916	M	Sp	A		oligotrophic snow-melt ponds with or without vegetation
18.	*Aedes* (*Ochlerotatus*) *intrudens* (Dyar, 1919)	M	Sp	A		temporary reservoirs in forests with dead leaves at the bottom, floodplains and grassy or snow-melt water pools
19.	*Aedes* (*Hulecoeteomyia*) *japonicus* (Theobald, 1901)	P	Sp	A	+	small natural and artificial water reservoirs like three holes, rock pools, vases, tins, drums, tires, buckets, breeding sites which are rich in organic matter
20.	*Aedes* (*Ochlerotatus*) *leucomelas* (Meigen, 1804)	M	Sp	A, Z		flooded meadows and reeded areas, snow-melt reservoirs on forest edges, slightly saline water, and slightly acid to alkaline waters
21.	*Aedes* (*Ochlerotatus*) *nigrinus* (Eckstein, 1918)	P	Sp	Z		flooded meadows in river valleys
22.	*Aedes* (*Ochlerotatus*) *nigripes* (Zetterstedt, 1838)	M	Sp	A, Z		snow-melt ponds, willow, and birch shrub marches, oligotrophic reservoirs, shallow puddles surrounded by *Carex* sp.
23.	*Aedes* (*Ochlerotatus*) *pionips* (Dyar, 1919)	M	Sp	A		snow-melt ponds in boggy forests
24.	*Aedes* (*Ochlerotatus*) *pullatus* (Coquillett, 1904)	M	Sm	Z		small clear water snow-melt reservoirs, boggy holes, water reservoirs without vegetation or with rocky bottom in mountainous regions
25.	*Aedes* (*Ochlerotatus*) *punctor* (Kirby, 1837)	P	Sp	Z		snow-melt ponds in swampy forests, meadows, boggy waters, acidic reservoirs with *Sphagnum* sp.
26.	*Aedes* (*Ochlerotatus*) *riparius* (Dyar and Knab, 1907)	M	Sp	A		peat bogs, mixed and deciduous forests, reservoirs with leaf debris on the bottom
27.	*Aedes* (*Aedes*) *rossicus* Dolbeskin, Gorickaja and Mitrofanowa, 1930	P	Sp	A	+	small temporary water bodies, inundation areas of river valleys, swampy woodlands with acid waters
28.	*Aedes* (*Ochlerotatus*) *rusticus* (Rossi, 1790)	M	Sp	A		snow-melt temporary forest reservoirs, partly shaded woodlands
29.	*Aedes* (*Ochlerotatus*) *sticticus* (Meigen, 1838)	M	Sm	A	+	river valleys, shaded flood reservoirs with neutral to alkaline waters
30.	*Aedes* (*Aedimorphus*) *vexans* (Meigen, 1830)	P	Sp	A, Z	+	inundation areas of river valleys and lakes, temporary water bodies with neutral to alkaline water, flooded meadows, poplar cultures, willow and reed areas
31.	*Anopheles* (*Anopheles*) *atroparvus* van Thiel, 1927	M	Sm	A, Z	+	sun exposed stagnant, semi-permanent, or permanent breeding sites, in saline and freshwater reservoirs, brackish water, canals, ditches, marshes, river margins, pools in riverbeds with a considerable amount of filamentous green algae and other floating and submerged vegetation
32.	*Anopheles* (*Anopheles*) *claviger* (Meigen, 1804)	P	Sp	A	+	ponds, tree hollows, ditches with clear water and reeds
33.	*Anopheles* (*Anopheles*) *daciae* Linton, Nicolescu & Harbach, 2004	P	Sp	A		stagnant clear reservoirs on the banks of rivers and lakes with abundant growth of submerged vegetation, swamps, flood plains, ponds, and ditches, larger water bodies on floodplains
34.	*Anopheles* (*Anopheles*) *hyrcanus* (Pallas, 1771)	P	Sp	A	+	sun-exposed stagnant water bodies on floodplain, water bodies rich in aquatic vegetation and vertical structures like reeds, swamps, rice fields, edges of slowly moving waters such as grassy streams, ditches, and canals, larvae tolerate a slight degree of salinity, can be found on coastal and inland marshes
35.	*Anopheles* (*Anopheles*) *maculipennis s.s.* Meigen, 1818	P	Sp	A, Z	+	meadows, the sheltered edges of floating streams with clear water, irrigation fields, calm rivers, small water bodies without vegetation and artificial collections of water, waters with a high content of organic matter
36.	*Anopheles* (*Anopheles*) *messeae* Falleroni, 1926	P	Sp	Z	+	stagnant clear reservoirs on the banks of rivers and lakes with abundant growth of submerged vegetation, swamps, flood plains, ponds, and ditches, larger water bodies on floodplains
37.	*Anopheles* (*Anopheles*) *petragnani* del Vecchio, 1939	P	Sm	Z		in fresh water rock pools in river beds or in ditches and drainage canals with alkaline, low conductivity and low values of hardness, with little vegetation also in artificial breeding sites like wells and cisterns
38.	*Anopheles* (*Anopheles*) *plumbeus* Stephens, 1828	P	Sp	A, Z	+	forests, edges of the forests, exclusively in tree hollows in dark brown waters with dissolved tannins and pigments also with high concentration of salts
39.	*Coquillettidia* (*Coquillettidia*) *richiardii* (Ficalbi, 1889)	M	Sm	A	+	permanent water reservoirs with rich vegetation
40.	*Culiseta* (*Culiseta*) *alaskaensis* (Ludlow, 1906)	P	Sp	A, Z		permanent meltwater reservoirs
41.	*Culiseta* (*Culiseta*) *annulata* (Schrank, 1776)	P	Sp	A, Z	+	ponds, ponds, ditches, brackish water, rainwater in vessels and waste
42.	*Culiseta* (*Culicella*) *fumipennis* (Stephens, 1825)	M	Sp	Z		ponds, wetlands, reservoirs with abundant vegetation, rarely brackish waters
43.	*Culiseta* (*Culiseta*) *glaphyroptera* (Schiner,1864)	P	Sp	Z		cool reservoirs with clear water, hollows of trees, depressions along streams
44.	*Culiseta* (*Culicella*) *morsitans* (Theobald, 1901)	M	Sp	Z	+	ponds, small temporary reservoirs, vessels, wet forests, brackish and saline waters
45.	*Culiseta* (*Culicella*) *ochroptera* (Peus, 1935)	M	Sp	Z	+	peat bogs, shallow swamps, forest ponds and ditches, lakes
46.	*Culiseta* (*Culiseta*) *subochrea* (Edwards, 1921)	P	Sp	A, Z		clear water ditches and ponds, shady brackish waters
47.	*Culex* (*Maillotia*) *hortensis* Ficalbi, 1889	P	Sp	Z		clear water with vegetation, unused wells, and vessels, small water reservoirs
48.	*Culex* (*Barraudius*) *modestus* Ficalbi, 1889	P	Sm	A	+	meadows, rice fields, brackish waters
49.	*Culex* (*Culex*) *pipiens s.l.* Linnaeus, 1758	P	Sm	A	+	polluted water reservoirs, underground stormwater drainage system (biotype *molestus*); almost any type of stagnant water (biotype *pipiens*)
50.	*Culex* (*Neoculex*) *territans* Walker, 1856	P	Sp	Z		lakes and ponds, swamps, areas along streams, polluted waters
51.	*Culex* (*Culex*) *torrentium* Martini, 1925	P	Sp	A	+	water tanks similar to *Cx*. *pipiens* biotype *pipiens*, but with clearer water

P—polycyclic; M—monocyclic; Sp—spring; Sm—summer; A—anthropophilic; Z—zoophilic.

## Data Availability

No new data were created or analyzed in this study. Data sharing is not applicable to this article.
